# Ce-based solid-phase catalysts for phosphate hydrolysis as new tools for next-generation nanoarchitectonics

**DOI:** 10.1080/14686996.2023.2250705

**Published:** 2023-09-08

**Authors:** Makoto Komiyama

**Affiliations:** Research Center for Advanced Science and Technology (RCAST), The University of Tokyo, Tokyo, Japan

**Keywords:** Phosphate hydrolysis, CeO_2_, metal-organic framework (MOF), nanoarchitectonics

## Abstract

This review comprehensively covers synthetic catalysts for the hydrolysis of biorelevant phosphates and pyrophosphates, which bridge between nanoarchitectonics and biology to construct their interdisciplinary hybrids. In the early 1980s, remarkable catalytic activity of Ce^4+^ ion for phosphate hydrolysis was found. More recently, this finding has been extended to Ce-based solid catalysts (CeO_2_ and Ce-based metal-organic frameworks (MOFs)), which are directly compatible with nanoarchitectonics. Monoesters and triesters of phosphates, as well as pyrophosphates, were effectively cleaved by these catalysts. With the use of either CeO_2_ nanoparticles or elegantly designed Ce-based MOF, highly stable phosphodiester linkages were also hydrolyzed. On the surfaces of all these solid catalysts, Ce^4+^ and Ce^3+^ coexist and cooperate for the catalysis. The Ce^4+^ activates phosphate substrates as a strong acid, whereas the Ce^3+^ provides metal-bound hydroxide as an eminent nucleophile. Applications of these Ce-based catalysts to practical purposes are also discussed.

## IMPACT STATEMENT

This review covers recent developments of synthetic catalysts to hydrolyze biorelevant phosphates, which are essential for constructing interdisciplinary hybrids between nanoarchitectonics and biology.

## GRAPHICAL ABSTRACT

**Figure:**
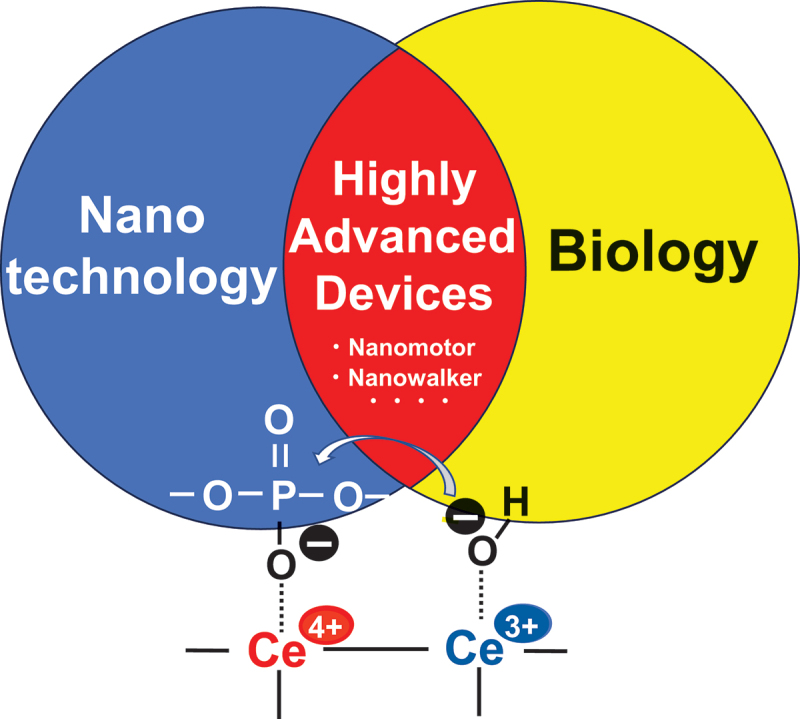

## 1. Introduction

Hybridizations of nanoarchitectonics and biology are enormously attractive since remarkable interdisciplinary synergisms are expected there [1–12]. Nanoarchitectonics provides sophisticatedly designed nanostructures which are otherwise hardly obtainable. When necessary, auxiliary groups for additional functions (including biomolecules) can be introduced to the required sites. On the other hand, biology is characterized by prompt and selective transformations of biomolecules without undesired byproducts. Strict molecular recognition between biomolecules is also a significant advantage. Accordingly, unprecedentedly advanced systems should be obtainable, when these two disciplines are combined so that multiple biological-transformations (or their chemical mimics) proceed on (or in) nanoarchitectures. In terms of this concept, various nanomachines were already constructed [5,12–38]. They involve nanomotors [21–25], nanorobots [26], nanocarriers of drugs [27–30], DNA walkers [20,31–33], ultrasensitive sensors [35–38], and others. In a rolling motor, for example, a DNA-modified silica nanoparticle directionally moves on a gold plate which is covered by RNA monolayer [24]. The driving force of the movement is the digestion of RNA by an enzyme RNase H, which digests the RNA in the DNA/RNA heteroduplex. The DNA on the nanoparticle forms a heteroduplex with the RNA on the plate, and induces the hydrolysis of this RNA by the RNase H. This enzymatic hydrolysis of RNA consecutively occurs on the surface of the gold plate, and autonomously moves the nanoparticle.

In terms of hybridization of nanoarchitectonics with biology, highly sophisticated and complicated nanomachines can be manipulated. The dynamic functions of these systems are mostly based on enzymatic cleavages of phosphates and esters, in which the product of a reaction controls the other reaction(s) as either substrate or modulator. As a result, multiple chemical reactions proceed in an ordered fashion with precise spatiotemporal control. Complicated networks of multiple enzymatic reactions can be straightforwardly obtained since unnecessary crosstalk between the reactions is avoidable in terms of both structural restraints in nanoarchitectonics and highly selective molecular recognition in biology. The resultant chemical and/or physical perturbations in the hybrids induce unprecedentedly sophisticated dynamisms. Most significantly, molecular design of these approaches is completely free according to our needs. Thus, biology/nanoarchitectonics interdisciplinary hybridization can go far beyond the current nanotechnology, leading to novel next-generation technology.

**Figure 1.**
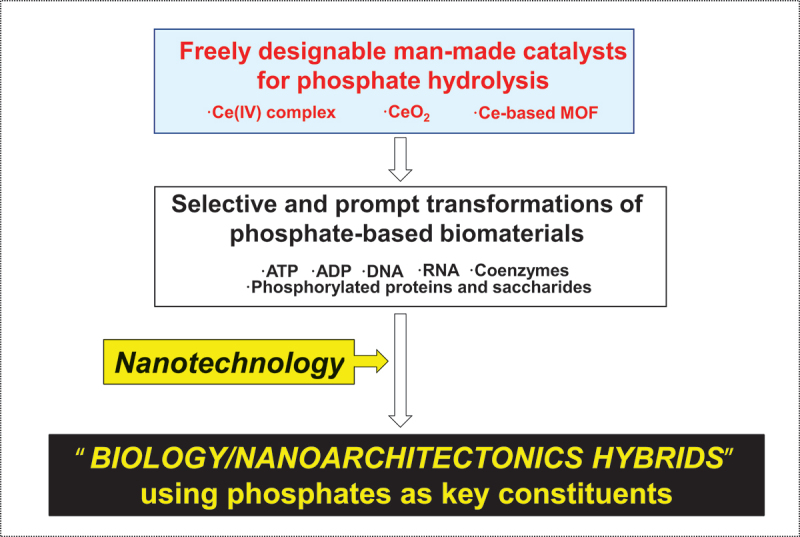
Developments of Ce-based catalysts for phosphate hydrolysis to construct next-generation biology/nanoarchitecture hybrids.

One of the most essential requirements for the construction of these interdisciplinary hybrids is catalysts which transform target biomolecules into others with sufficient efficiency and selectivity. However, direct incorporation of naturally occurring enzymes into nanostructures is often troublesome and unacceptable, because of their large molecular size, limited substrate-specificity, and intrinsic instability. Chemistry-based catalysts (enzyme mimics) are far more desirable since their properties are freely tunable according to our need. In many cases, solid-phase catalysts are especially convenient for constructing these hybrids. This review focuses onto synthetic catalysts for the hydrolysis of biorelevant phosphates, which are ubiquitous and take inevitable roles in biology (Figure 1). For example, adenosine triphosphate (ATP) serves as the main energy source for most bioreactions, whereas DNA stores genetic information. RNA shows various unique functions in terms of its flexible structure [5,15,16]. Many coenzymes involve phosphate or pyrophosphate linkages (e.g. nicotinamide adenine dinucleotide and thiamine pyrophosphate). On the other hand, phosphorylation/dephosphorylation of proteins (serine, threonine, and tyrosine residues) regulates the activities of these proteins, leading to intravital dynamic motions, controlled information transmission, switching of protein/protein interactions, and many other complicated biofunctions [39–41]. Thus, elegant interdisciplinary hybrids can be fabricated by precisely placing phosphatase mimics with biorelevant phosphate compounds at predetermined positions in nanoarchitectures. When necessary, various functional molecules can be additionally incorporated into the desired sites of the conjugates for more complicated actions. These hybrids can be further connected to each other in still larger and more advanced assemblies.

## 2. Developments of catalysts for phosphate hydrolysis

**Figure 2.**
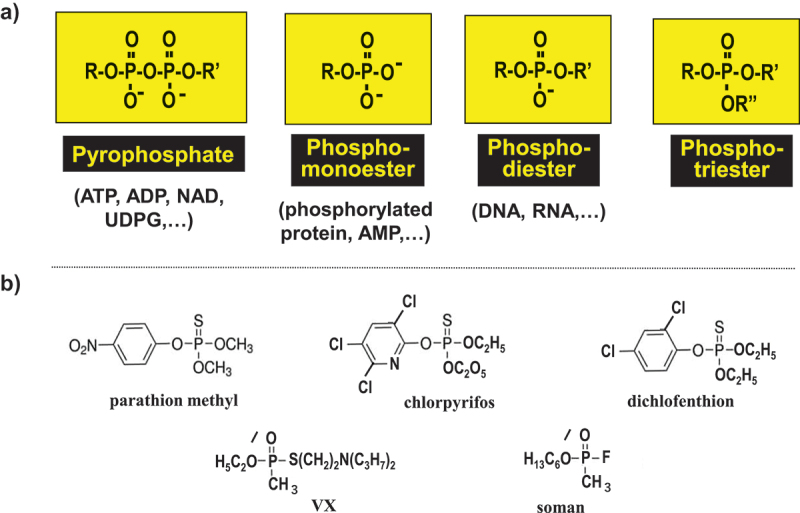
(a) Structures of four types of phosphate compounds which are hydrolyzed by Ce-based solid catalysts in this review. Typical biocompounds are presented in parentheses. However, phosphorylation in our body is never restricted to these compounds, and occurs very often to accomplish variety of inevitable roles. In (b), pesticides and nerve gases, decomposed by Ce-based solid catalysts in 5.1, are presented.

In most cases, the reactivities of phosphates for hydrolytic scission are in the following order; pyrophosphates >> phosphomonoesters > phosphotriesters >> phosphodiesters (their structures are presented in Figure 2(a)). Various metal complexes have already been developed to hydrolyze pyrophosphates and phosphomonoesters efficiently, and phosphotriesters at moderate rates [42–58]. On the other hand, phosphodiesters were far more stable than them, and hardly hydrolyzed by artificial catalysts for a long time (only activated linkages in bis(4-nitrophenyl) phosphate and supercoiled plasmid DNA were hydrolyzed). Apparently, still more active catalysts are required to construct biology/nanoarchitecture hybrids.

About 20 years ago, a remarkable catalysis of CeCl_3_ for the hydrolysis of phosphodiester linkages in DNA was discovered by the author’s group [59,60]. It was soon shown that Ce^4+^ ions, formed through in situ oxidation of the Ce^3+^ ions, are responsible for the catalytic hydrolysis [55,60–62]. Nowadays, Ce(NH_4_)_2_(NO_3_)_6_ is usually used as catalytic agent [63]. Compared with Ce^4+^, other lanthanide(III) ions (including Ce^3+^) and non-lanthanide ions are far less active. Zr^4+^ and Th^4+^ are effective only in acidic solutions [64,65]. For the hydrolysis of phosphomonoesters, phosphotriesters, and pyrophosphates, Ce^4+^ is also overwhelmingly more active than the other metal ions [66–69]. Many water-soluble complexes of this tetravalent ion were already reported [70–84].

More recently, various Ce-based solid catalysts (CeO_2_ (ceria) and Ce-based metal-organic frameworks (MOFs)) have been developed for the hydrolysis of phosphates (pyrophosphates and three types of phosphoesters), as discussed in this review. These catalysts are conveniently applicable to biology/nanoarchitecture hybrids, which should lead to wide applications in biochemistry, medicine, pharmaceutics, informatics, and many other fields. Furthermore, the features of these catalysts are precisely modulated by sophisticated engineering. In Section 3 of this review, the catalytic mechanisms of Ce^4+^ for phosphate hydrolysis in aqueous phase, as well as cooperative promotion of Ce^4+^ catalysis by other metal ions, are briefly described as the fundamental information on Ce-based solid catalysts. Sections 4 and 5 present successful examples of CeO_2_ catalysts and MOF catalysts, respectively. In Section 6, recent progresses in practical applications of Ce-based catalysts are described. Among various metal ion catalysts, Ce is characterized by the stability of both +3 and +4 states, and these two states are easily converted into each other under mild conditions. Throughout this review, the critical importance of the cooperation of Ce^4+^ and Ce^3+^ ions for the efficient catalysis is emphasized.

## 3. Mechanism of Ce^4+^ catalysis for phosphate hydrolysis

After the discovery of remarkable catalysis of Ce^4+^ for the hydrolysis of phosphates in aqueous solutions, its catalytic mechanism was the primary concern [45]. Especially, its catalysis for DNA hydrolysis was investigated in detail. Highly important roles of cooperation of two Ce^4+^ ions were evidenced. Furthermore, the catalytic activity of Ce^4+^ was greatly enhanced by combining this with another metal ion. In this section, these results are briefly overviewed as the fundamental information on Ce-based solid catalysts for phosphate hydrolysis.

### 3.1. Catalytic mechanism of Ce^4+^ ion

**Figure 3.**
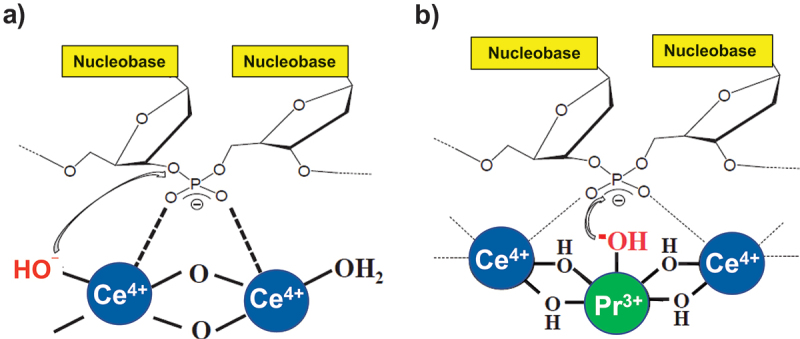
Proposed mechanisms of DNA hydrolysis in aqueous solutions (a) by Ce^4+^ ion and (b) by Ce^4+^/Pr^3+^ combination. In (a), two Ce^4+^ ions in bimetallic cluster [Ce_2_(OH)_4_]^4+^ activate the phosphodiester as acid catalysts, and one of them provides metal-bound hydroxide as nucleophile. In (b), two Ce^4+^ ions in Ce^4+^/Pr^3+^ 2:1 bimetallic cluster activate the phosphodiester linkage, and Pr^3+^ provides its metal-bound hydroxide as nucleophile. Note that Pr^3+^-bound hydroxide is far better nucleophile than Ce^4+^-bound hydroxide.

Ce^4+^-catalyzed DNA hydrolysis in aqueous solutions was kinetically analyzed under acidic conditions (pH < 2.5), where Ce^4+^ hardly forms hydroxide gel [85,86]. In the reaction mixtures, Ce^4+^ ions are distributed into various species (Ce^4+^, [Ce(OH)]^3+^, [Ce(OH)_2_]^2+^, [Ce_2_(OH)_4_]^4+^, and others). It was shown that the catalytically active species for DNA hydrolysis is a bimetallic hydroxo-cluster [Ce_2_(OH)_4_]^4+^, in which two Ce^4+^ ions are connected by two bridging OH groups (Figure 3(a)). This structure was supported by an extended X-ray absorption fine structure study [87]. Note that all the tetravalent actinides (Th^4+^, U^4+^, and Pu^4+^) also form similar bimetallic clusters [88].

In a proposed mechanism [85,86], the scissile phosphodiester linkage is first coordinated to the two Ce^4+^ ions in [Ce_2_(OH)_4_]^4+^. As a result, the electrons in the phosphodiester are strongly withdrawn, activating the linkage for the nucleophilic attack. Mixing of the 4f orbitals of Ce^4+^ with the orbitals of the phosphodiester promotes this activation [89]. The phosphorus atom is then attacked by a hydroxide ion which is coordinated to one of the two Ce^4+^ ions. Although this metal-bound hydroxide is a poor nucleophile, the following three factors allow the reaction to proceed promptly. First, the phosphodiester is sufficiently activated by the dual acid catalysis of two Ce^4+^ ions in [Ce_2_(OH)_4_]^4+^. Secondarily, the Ce^4+^-bound hydroxide is located in a suitable position for intramolecular nucleophilic attack. Finally, the positive charges, accumulated in [Ce_2_(OH)_4_]^4+^ cluster, electrostatically stabilize the negatively charged transition state of DNA hydrolysis. In the breakdown of the resultant penta-coordinated intermediate, the water bound to a Ce^4+^ ion in the cluster functions as an acid catalyst to facilitate the removal of the very unstable 5’-alkoxide ion of 2’-deoxyribonucleotide from the phosphorus atom.

### 3.2. Cooperation of Ce^4+^ with another metal ion to promote phosphate hydrolysis

In many of naturally occurring enzymes for transformation of phosphates (alkaline phosphatase, purple acid phosphatase, and others), two or more metal ions (e.g. Mg^2+^, Zn^2+^, and Mn^2+^) share the roles as acid catalyst(s) and base catalyst(s) for efficient catalysis [90–92]. Interestingly, a similar bimetallic cooperation was also evidenced in non-enzymatic systems [93–97]. For example, Ce^4+^-catalyzed hydrolysis of DNA (in Figure 3(a)) was promoted by more than 10-fold, when Pr^3+^ (or Nd^3+^) ions coexisted as additive [93]. For DNA hydrolysis, Pr^3+^ itself (or Nd^3+^ itself) is inactive. Kinetic study indicated that 2:1 mixed hydroxide cluster is formed from Ce^4+^ and Pr^3+^ (see Figure 3(b)). In the catalysis, the scissile phosphodiester linkage of DNA is coordinated to the two Ce^4+^ ions in the cluster. As a result, the electrons on the P atom are drastically withdrawn by these two Ce^4+^ ions to activate the reaction center. On the other hand, the Pr^3+^ ion in the cluster provides its metal-bound hydroxide as the nucleophile for the reaction. In the mixed cluster, the Pr^3+^ and Ce^4+^ ions are located nearby for the efficient cooperation. In the DNA hydrolysis by Ce^4+^ alone in Figure 3(a), however, the nucleophile is provided by one of the Ce^4+^ ions. Note that the Pr^3+^-bound hydroxide is much better nucleophile than Ce^4+^-bound hydroxide (the pK_a_ value of Pr^3+^-bound water is >7, whereas the corresponding value for Ce^4+^ is <1 [98,99]). This difference is primarily responsible for the far larger catalytic activity of Ce^4+^/Pr^3+^ combination than Ce^4+^ alone.

Similar bimetallic cooperation for DNA hydrolysis was also remarkable, when La^3+^ was combined with Sn^4+^ or Fe^3+^ [94,95]. Without the cooperation, all these metal ions are inactive for DNA hydrolysis. In addition to DNA hydrolysis, eminent catalysts for RNA hydrolysis were also obtained by combining two kinds of metal ions (e.g. Sn^4+^/La^3+^, Fe^3+^/La^3+^, Sn^4+^/Zn^2+^, and Al^3+^/Zn^2+^ combinations). Furthermore, dinuclear coordination complexes involving two different metal ions (e.g. Fe^3+^/Zn^2+^ and Fe^3+^/Cu^2+^) were fabricated for phosphate hydrolysis [91,100]. In metal-organic frameworks (MOFs), Ti^4+^/Fe^3+^ cooperation (for the hydrolysis of diisopropyl fluorophosphate) [96] and Ti^4+^/Ca^2+^ cooperation (for cycloaddition) [97] were evidenced. It is noteworthy that, as described in the following sections, analogous cooperative catalysis occurs between Ce^4+^ and Ce^3+^ in Ce-based solid catalysts, and is the main origin of their enormous catalytic activity.

## 4. Catalysis of CeO_2_ (ceria) for the hydrolysis of phosphoesters and pyrophosphates

In 2008, it was discovered that CeO_2_ nanoparticles efficiently remove a phosphate group from tyrosine (Y), threonine (T), and serine (S) in phosphopeptides [101]. These hydrolyses of phosphomonoesters were very fast. For example, the dephosphorylation from pY (phospho-L-tyrosine) and pT (phospho-L-threonine) in FLTE-pY-VATR or FL-pT-EYVATR was completed within 10 min, when the phosphorylated nonapeptides were treated with commercially obtained ceria powder (1–10 μm diameter) at 37°C in 0.1 M ammonia solution. This dephosphorylation was employed for peptide mass mapping [102]. Later, the catalysis of ceria for the hydrolysis of various phosphoesters was evidenced [103–105]. These findings have greatly widened the scope of practical applications.

There are a variety of methods to synthesize nanoparticles of CeO_2_ (precipitation, hydrothermal, microemulsion, and others), as clearly summarized in a review [106]. More environment-friendly nanoparticles are obtainable by using natural matrices as stabilizing agents [107]. Very importantly, the catalytic activities of CeO_2_ for phosphate hydrolysis (also for other reactions) are strongly dependent on the preparation method, reflecting the difference of the obtained CeO_2_ in shape, size, surface area, crystallinity, defects, charges, and other physicochemical features.

### 4.1. Hydrolysis of phosphomonoesters and pyrophosphates by CeO_2_

**Figure 4.**
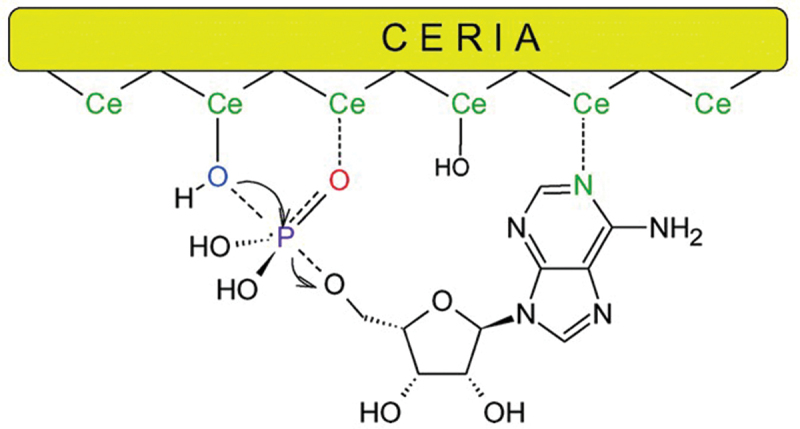
Catalysis of CeO_2_ for the hydrolysis of AMP as one of the pioneering works in the field. In addition to the cooperative catalysis of two Ce ions, the interaction of another Ce with adenine moiety is proposed. Note that one (or two) of the OH groups on the phosphorus atom is probably ionizing under the reaction condition (pH 7.22). Reproduced from Ref. [104]. With permission from the Royal Society of Chemistry.

In aqueous solutions, various monophosphates and pyrophosphates were efficiently hydrolyzed by CeO_2_ [103–105]. In Figure 4, adenosine monophosphate (AMP) was converted to adenosine and H_3_PO_4_. The CeO_2_ catalysts were prepared by precipitating Ce(NO_3_)_3_ solution with NH_4_HCO_3_, followed by annealing of the resultant cerium carbonate precursor. With the use of 2.0 g of this CeO_2_ in 100 mL (pH 7.22 Tris buffer), more than 50% of AMP was hydrolyzed within 10 min at 22°C [104]. The pyrophosphate linkages in ATP and ADP (adenosine diphosphate) were also rapidly cleaved by CeO_2_ catalysts. These reactions are completely hydrolytic, although most of previous catalyses of CeO_2_ involve oxidation/reduction of substrates [108–110]. Commercially obtained CeO_2_ particles were also employable as catalysts for these dephosphorylations. Very small-sized particles (4 nm diameter), prepared by stirring NaOH solution of Ce(NO_3_)_3_ at 700 rpm for 22 h at 25°C, showed still higher activity, as expected [105]. Fiber- and nanorod-structured CeO_2_ was also effective for the catalysis [111,112]. According to X-ray photoelectron spectroscopy analysis, there exist considerable amounts of Ce^3+^ ions, in addition to Ce^4+^, on the surface of CeO_2_ through redox reactions (Ce^3+^ ⇄ Ce^4+^). The resultant oxygen vacancies are the active sites for the catalysis [113–115]. It was proposed that these Ce^4+^ and Ce^3+^ ions cooperate with each other for the catalysis, as discussed in Section 3.2. More acidic Ce^4+^ activates the phosphate through the complex formation, whereas the adjacent Ce^3+^ provides metal-bound hydroxide as the nucleophile. Note that Ce^3+^-bound hydroxide ion is far stronger nucleophile than Ce^4+^-bound hydroxide ion (the pK_a_ value of Ce^3+^-bound water is much higher than the value of Ce^4+^ [98,99]). Accordingly, the cooperation of Ce^3+^ remarkably promotes the catalytic activity of Ce^4+^. Essential roles of Ce^4+^/Ce^3+^ couples in CeO_2_ have also been confirmed in its catalysis for oxidation, hydrogenation, and other reactions [116,117].

On the hydrolysis of 4-nitrophenyl phosphate (an activated phosphomonoester having a good leaving group), the dependence of catalytic activity on the structure of CeO_2_ was analyzed in detail. The order of activity was as follows: nano-octahedra > nanorods > nanocubes [103,105,115]. On the surface of nano-octahedra, (111) facet is preferentially exposed. On the other hand, both (110) and (100) sites are dominant on the nanorods, whereas (100) site is abundant on the nanocubes. Thus, the catalytic activities are (111) > (110) > (100), which is consistent with the order of the Lewis acidity of surface [113,118,119]. On (111) surface, Ce atoms are 7-coordinated with one vacant orbital, whereas (110) and (100) surfaces involve 6-coordinated Ce atoms with two vacant orbitals. Thus, the (111) surface is the most Ce^4+^-like [119–121]. Interestingly, (111) crystal face is also responsible for the catalysis in CeO_2_-mediated hydrolysis of 4-methyl-1,3-dioxane to 1,3-butanediol [122]. The relationship between the kind of crystalline facet of CeO_2_ and its catalytic activity was discussed in detail for the catalysis of oxidation reactions in a review [123].

Unfortunately, these CeO_2_ catalysts, prepared by conventional methods, do not hydrolyze phosphotriesters and phosphodiesters [104], which are less reactive than phosphomonoesters and pyrophosphates. In order to hydrolyze them, more sophisticated engineering of CeO_2_ is necessary, as described in the following two sections.

### 4.2. Hydrolysis of phosphotriesters by valency-engineered CeO_2_

**Figure 5.**
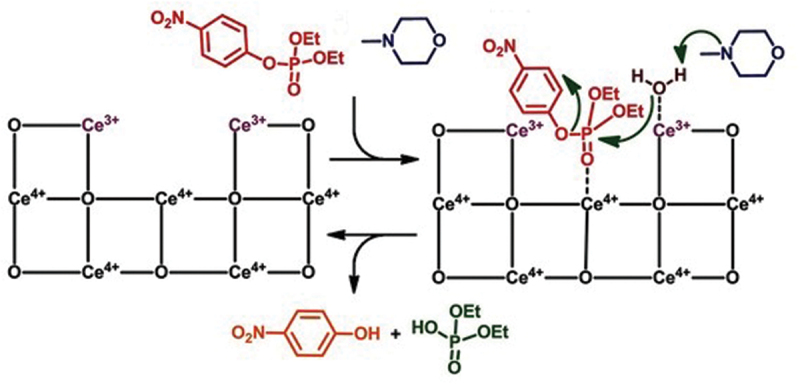
Hydrolysis of diethyl 4-nitrophenyl phosphate by vacancy-engineered CeO_2_. Reproduced from Ref. [124]. With permission from John Wiley and Sons.

Phosphotriesters are more stable than monophosphates and pyrophosphates and hardly hydrolyzed by conventional CeO_2_. Smaller electrostatic interactions of phosphotriesters with positively charged Ce ion are primarily responsible for this feature. Accordingly, some special methodologies have been employed to obtain more active CeO_2_. One of them is to add H_2_O_2_ during the synthesis of CeO_2_ to engineer the valences of Ce ions therein, and increase the number of active sites on the surface [124,125]. Typically, CeCl_3_ (100 mg) was dissolved in deionized water (15 mL), and 0.5 mL H_2_O_2_ (30%) and 1.5 mL NH_4_OH (30%) were added. After keeping the mixture at 100°C, the engineered CeO_2_ was obtained as a yellow precipitate. Simply with the use of this valency-engineered CeO_2_, however, even diethyl 4-nitrophenyl phosphate (paraoxon; an activated phosphotriester) was not hydrolyzed. In order to hydrolyze this phosphotriester, simple incorporation of Ce^3+^ is insufficient. Notable hydrolysis occurred, only when N-methylmorpholine was furthermore added to the mixture (Figure 5). In the catalysis, the phosphotriester is first activated by the coordination to the Ce^4+^ sites. The water at the Ce^3+^ site then attacks its phosphorus center as nucleophile. Here, N-methylmorpholine acts as general-base catalyst and activates Ce^3+^-bound water through the abstraction of a proton from this water. The essential role of Ce^3+^/Ce^4+^ cooperation for the present catalysis was confirmed by the following two experiments. First, when the engineered CeO_2_ was pretreated with KIO_4_ to oxidize most of the Ce^3+^ to Ce^4+^, the catalytic hydrolysis of diethyl 4-nitrophenyl phosphate hardly occurred even in the presence of N-methylmorpholine. Secondarily, the pretreatment of the engineered CeO_2_ with ascorbic acid (or NaBH_4_) to reduce Ce^4+^ to Ce^3+^ also diminished the catalytic activity.

Alternatively, phosphotriester-hydrolyzing CeO_2_ was prepared by replacing some of Ce^4+^ with Zr^4+^ to create catalytic vacancies there [126–128]. In water, CeCl_3_ and ZrOCl_2_ were dissolved, and NH_4_OH (30%) was added. The mixture was incubated at 100°C until yellow colored dispersion of Zr-doped CeO_2_ was obtained. As a result, Ce^4+^/Ce^3+^ pairs were generated near the Zr^4+^ ions. In the presence of N-methylmorpholine as general-base catalyst, the Zr-incorporated CeO_2_ hydrolyzed dimethyl 4-nitrophenyl phosphate. In contrast, the CeO_2_ prepared in the absence of ZrOCl_2_ was inactive for the catalysis, even in the presence of a general base catalyst.

Another methodology to hydrolyze phosphotriesters is to control the shape of CeO_2_ and expose catalytically active (111) crystal face on its surface (this crystal face is also the most suitable for the hydrolysis of phosphomonoesters, as described in 4.1). For example, CeO_2_ nanooctahedra (6–35 nm size), which dominantly expose (111) crystal faces, cleaved diethyl 4-nitrophenyl phosphate (paraoxon) with sufficient efficiency, even in the absence of N-methylmorpholine [129,130]. Submicron-sized octahedra (150–220 nm size; (111) faces dominant) were also active. On the other hand, nanocubes (5–60 nm), dominated by (100) crystal faces, showed only marginal activity. Alternatively, water-soluble CeO_2_ nanoparticles hydrolyzed diethyl 4-nitrophenyl phosphate without general-base catalyst [131]. To an aqueous solution of Ce(NO_3_)_3_, β-cyclodextrin was added, and then NaOH solution was introduced. In this procedure, the surface of CeO_2_ nanoparticles (32 nm diameter) was covered with β-cyclodextrin molecules. CeO_2_ nanoparticles (5 nm diameter), embedded in *N*-doped carbon material (substrate-binding site), also hydrolyzed diethyl 4-nitrophenyl phosphate [132].

### 4.3. Hydrolysis of phosphodiesters by CeO_2_ nanoparticles

The phosphodiester linkages in DNA are enormously resistant against hydrolytic scission, as described above. In 2019, however, it was found that these linkages are hydrolyzed at reasonable rates by commercially obtained CeO_2_ nanoparticles of 5 nm diameter [133]. These nanoparticles are one of ‘nanozymes’ which are characterized by large catalytic activity and specificity [134–140]. When a single-stranded oligonucleotide A_90_ (composed of 90 units of 2’-deoxyadenosine) was incubated with these CeO_2_ nanoparticles (100 mg/L) at 60°C for 1 h, almost all of the substrate molecules were converted into shorter fragments. The major product was A_5_, which was accompanied by smaller amounts of longer fragments (<15-nucleotide). Apparently, the catalytic scission was efficient only for sufficiently long DNA, and marginal for a DNA which is shorter than 5-nt length. The catalytic rate of DNA hydrolysis was strongly dependent on the temperature and showed a large jump between 40°C and 50°C. Double-stranded DNA was also cleaved, although the scission was by several fold slower than single-stranded DNA. There was no specific sequence-preference, and no oxidation of DNA occurred as confirmed by mass spectroscopy on the scission products. Very importantly, only small CeO_2_ particles (5 nm diameter) were effective for the DNA hydrolysis, and no catalysis was detected for the CeO_2_ particles of 165 nm diameter. This result is consistent with the cooperative catalysis of Ce^3+^/Ce^4+^ pairs since they are abundant only on the surface of small nanoparticles. In large particles, most of the Ce ions take the tetravalent state. Nanoparticles of other metal oxides (e.g. ZnO, TiO_2_, and MnO_2_) are almost completely inactive, and the activities of ZrO_2_ and In_2_O_3_ are much smaller.

**Figure 6.**
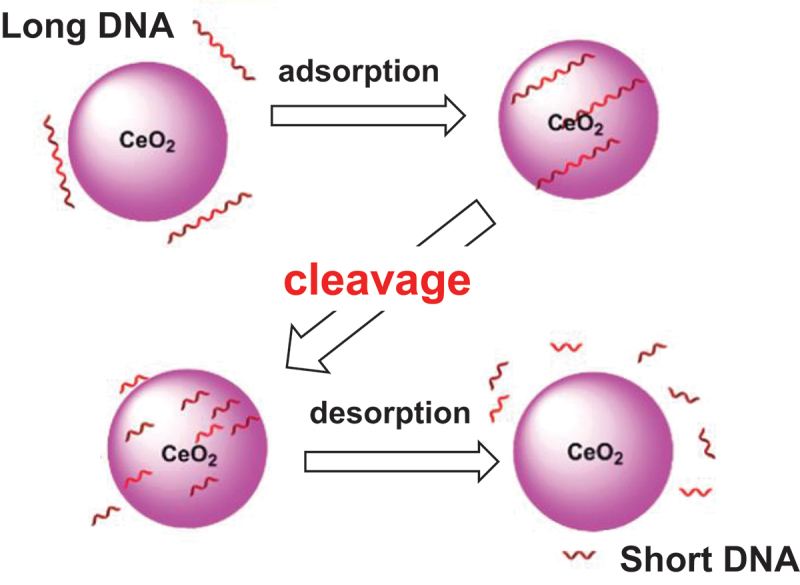
Cleavage of single-stranded DNA by CeO_2_ nanoparticles (5 nm diameter). Long DNA is effectively adsorbed by the particles, and then subjected to the catalysis. However, short DNA (<5-nucleotide) is hardly adsorbed and remains intact. Reproduced from Ref. [133]. With permission from the Royal Society of Chemistry.

The catalytic DNA hydrolysis by the small CeO_2_ nanoparticles starts with the adsorption of DNA substrate through the binding of the phosphate backbone to the Ce ions (Figure 6) [140]. These polyvalent interactions of DNA with CeO_2_ are necessary for the DNA to be activated for the hydrolysis. Accordingly, the catalysis is efficient only for the DNA of sufficient length. The catalytic efficiency decreases when the substrate is cleaved to shorter fragments. Very short DNA (<5-nucleotide) is not bound to the CeO_2_ nanoparticles and hardly hydrolyzed.

## 5. Cerium-based metal–organic frameworks (MOFs)

In metal–organic frameworks (MOFs), inorganic units are linked by organic units through coordinative bondings. They are characterized by multiple metal centers of well-defined structures, which are spatially arranged with a strict precision [141–145]. Their physicochemical features can be flexibly modulated in terms of sophisticated molecular design [146–148]. Accordingly, MOFs are promising candidates of superb enzyme mimics and industrial catalysts [149–152]. By introducing eminent Ce^4+^ (and/or Ce^3+^) ions to MOF, various efficient catalysts for phosphate hydrolysis have been constructed.

### 5.1. Hydrolysis of pyrophosphates by Ce-based MOF

**Figure 7.**
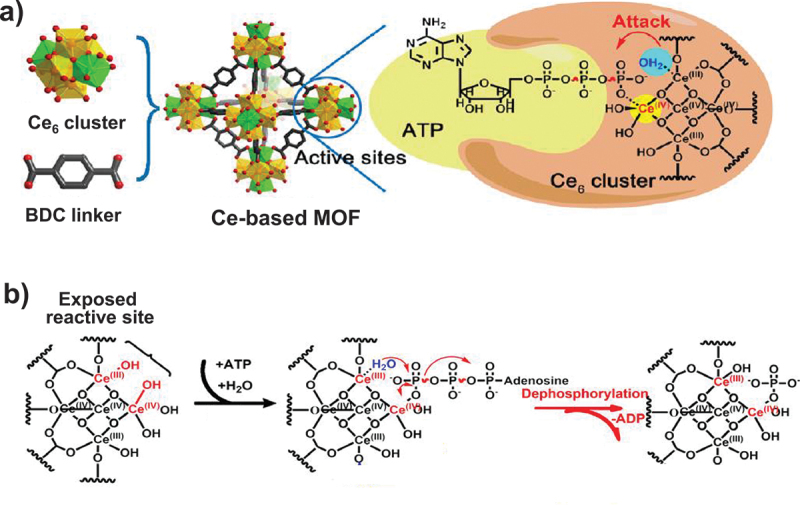
(a) Preparation of Ce-based MOF for the hydrolysis of ATP. (b) Proposed mechanism of catalysis by the Ce_6_ cluster in the MOF which involves the cooperation of Ce^4+^ and Ce^3+^. Reproduced from Ref. [153]. With permission from John Wiley and Sons.

A MOF (UiO-66(Ce)) in Figure 7(a) was prepared by incubating 1,4-benzenedicarboxylic acid (BDC) and Ce(NH_4_)_2_(NO_3_)_6_ at 80°C in 1:1 mole ratio with acetic acid modulator in DMF/water mixture [153]. The UiO-66(Ce) powder was activated in vacuum at 65°C. In this Ce-based MOF, Ce_6_O_4_(OH)_4_ clusters are stably connected by BDC units [154,155]. This MOF effectively hydrolyzes ATP into AMP and inorganic phosphate under physiological conditions. The catalytically active Ce_6_ clusters on the surface of MOFs involve Ce^3+^ ions and Ce^4+^ ions in about 1:2 ratio, as estimated by XPS analysis. Accordingly, each Ce_6_O_4_(OH)_4_ cluster contains two Ce^3+^ ions and four Ce^4+^ ions on average. These Ce^3+^ and Ce^4+^ ions cooperate for the pyrophosphate hydrolysis (Figure 7(b)) [156], as discussed above on the catalysis by CeO_2_. The Ce^4+^ ions bind the pyrophosphate group to promote its reactivity, whereas the Ce^3+^ ions provide their metal-bound water (probably in the form of its hydroxide) as the nucleophile for the hydrolysis.

Similarly, ADP was efficiently hydrolyzed to AMP and inorganic phosphate by this Ce-based MOF [153]. In Section 6.4, this catalysis is used to construct blood-compatible surface through the decomposition of ADP, which stimulates platelet aggregation as a signaling molecule.

### 5.2. Hydrolysis of monophosphates

The Ce-based MOF, prepared from Ce(NH_4_)_2_(NO_3_)_6_ and fumaric acid, efficiently hydrolyzed the monophosphate linkage in AMP [157]. This MOF was also active for the hydrolysis of pyrophosphate linkages in ATP and ADP. The phosphomonoesters in various phosphopeptides were removed by another UiO-66 MOF which was synthesized from 1/1 Ce(NH_4_)_2_(NO_3_)_6_/ZrO(NO_3_)_2_ mixture and 1,4-benzenedicarboxylic acid [158].

### 5.3. Preparation of advanced MOF for DNA hydrolysis

**Figure 8.**
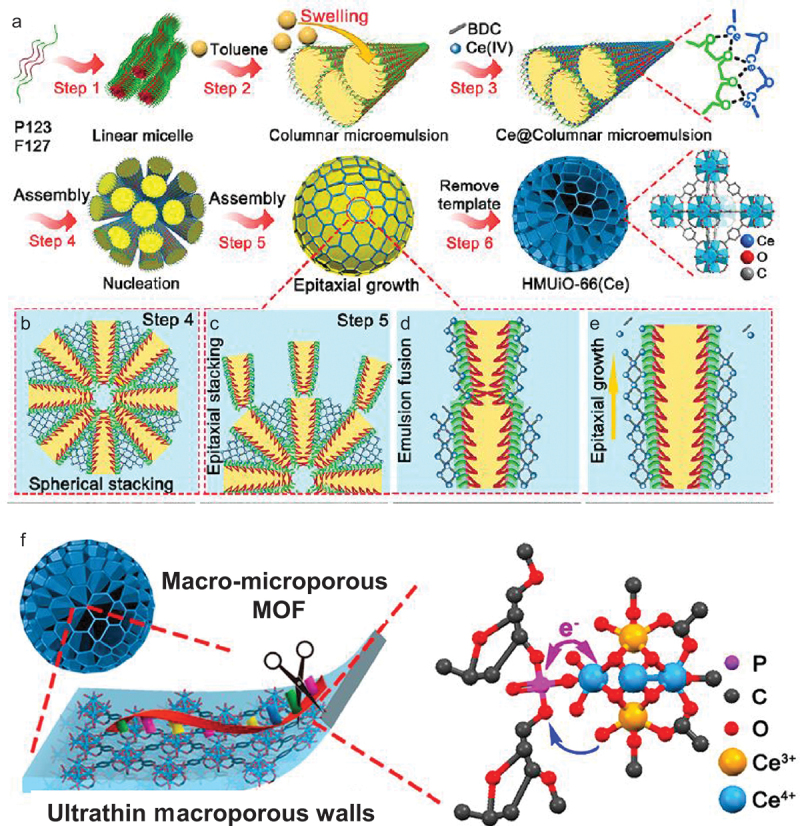
Preparation procedure of Ce-based macro-microporous MOF (a–e) and its catalytic mechanism for DNA hydrolysis (f). Reproduced from Ref. [159]. With permission from the American Chemical Society.

Stable phosphodiester linkages in DNA are not hydrolyzed by MOFs, which are synthesized by a conventional method. In order to hydrolyze DNA, the number of active site on the surface of MOF must be increased, and at the same time the intrinsic activity of each site should be enhanced. Furthermore, DNA of large molecular size must smoothly diffuse to the active site. In order to fulfill all these requirements, hierarchically macro-microporous MOFs were constructed with the use of microemulsion-guided assembly strategy (Figure 8(a–e)) [159]. In step 1, linear micelles were formed in the aqueous phase from P123 (a symmetric triblock copolymer comprising poly(ethylene oxide) and poly(propylene oxide)) and F127 (another triblock copolymer of a similar structure). These triblock polymer surfactants guided the growth of MOFs into ordered mesostructures [160,161]. Then, the micelle solution was transformed into microemulsion by adding toluene (step 2). In step 3, Ce(NH_4_)_2_(NO_3_)_6_ and BDC (1,4-benzenedicarboxylic acid) are added to form crown-ether-type complexes of Ce^4+^ with the poly(ethylene oxide) segments of P123. The MOFs crystallized along the anchored Ce clusters and assembled into a dendritic macroporous structure (step 4). Finally, open and continuous macrochannels with thin walls were formed (steps 5 and 6). The MOFs obtained possess macropores of 100 nm size for efficient diffusion of DNA substrates, and macroporous walls of 6–11 nm thickness. On these macroporous walls, there exist two types of micropores of 0.8 and 1.3 nm sizes which are responsible for the catalysis of DNA hydrolysis.

Exactly as designed, this MOF promptly hydrolyzed single-stranded DNA. With the use of this MOF (350 mg/L) at pH 7.4°C and 60°C, for example, almost all of 88-nt single-stranded DNA disappeared within 6 h. About 70% of plasmid DNA (form I) was converted to relaxed form II within 24 h. Abundantly exposed Ce^4+^-OH sites on macroporous walls are strongly coordinated with the scissile phosphodiester bond of DNA (Figure 8(f)). The adjacent Ce^3+^ sites work synergistically to provide a metal-bound hydroxide as the nucleophile, leading to hydrolytic cleavage of DNA. Apparently, highly accessible and exposed active sites in open spaces of this MOF are essential for the catalysis.

### 5.4. Catalytic MOFs prepared from Ce^3+^ salt

Using Ce(NO_3_)_3_ as the starting material in place of Ce(NH_4_)_2_(NO_3_)_6_, catalytically active MOF catalysts were prepared. In one of them, Ce(NO_3_)_3_ was mixed with 1,4-benzenedicarboxylic acid in DMF/H_2_O mixture, followed by heating at 85°C [162]. This MOF has a robust ladder-like supramolecular network, and hydrolyzed 4-nitrophenyl phosphate (an activated phosphomonoester) to 4-nitrophenol and inorganic phosphate. On the other hand, another MOF, prepared from Ce(NO_3_)_3_ and tetrakis(4-carboxyphenyl)ethylene at 120°C, hydrolyzed bis(4-nitrophenyl) phosphate (an activated phosphodiester) [163]. In the mechanisms proposed by the authors, Ce^3+^ ions are directly responsible for the catalysis. In another plausible mechanism, however, Ce^4+^ ions are formed in the preparation of these MOF catalysts, and cooperate with the Ce^3+^ ions for these catalyses, as discussed many times in this review.

## 6. Practical applications of phosphate hydrolysis by Ce-based catalysts

In this section, typical applications of Ce-based catalysts for the hydrolysis of phosphates and pyrophosphates for practical purposes are described. In many cases, CeO_2_ or MOF is employed, but Ce^4+^ complexes are also used according to the need.

### 6.1. Degradation of pesticides and chemical warfare agents by Ce-based solid catalysts

Many pesticides and chemical warfare agents are analogous to phosphates in structures (Figure 2(b)). The thiophosphate linkages in organophosphate pesticides (parathion methyl, chlorpyrifos, and dichlofenthion) were hydrolyzed by CeO_2_ [164,165]. Degradations of dangerous nerve gases VX (O-ethyl S-[2-(diisopropylamino) ethyl] methylphosphonothioate) and soman (O-pinacolyl methylphosphonofluoridate) were also notable. These efficient catalyses are ascribed to the cooperation of Ce^3+^/Ce^4+^ pair at the defects in CeO_2_ [166]. Furthermore, the processes of CeO_2_-mediated detoxification were directly followed by using Ce-doped carbon dots, which were fabricated from Ce(NO_3_)_3_ and EDTA by one-step hydrothermal carbonization. Upon degradation of chlorpyrifos, for example, the fluorescence intensity was gradually decreased due to the inner filter effect of the released chromophore (3,5,6-trichloro-2-pyridinol) [167].

A Ce^4+^-based MOF was prepared by incubating Ce(NH_4_)_2_(NO_3_)_6_ and 1,4-benzenedicarboxylic acid in DMF/water mixture at 100°C and rapidly hydrolyzed a nerve agent soman (CH_3_P(=O)(OC_6_H_13_)F) [168,169]. Interestingly, the catalytic activity was further promoted by combining the MOF with polyethyleneimine, which acts as a general-base catalyst in the same way as N-methylmorpholine in Figure 4. Zr^4+^-based MOF was also effective for detoxification [170].

### 6.2. Activation of prodrugs through dephosphorylation by CeO_2_

**Figure 9.**
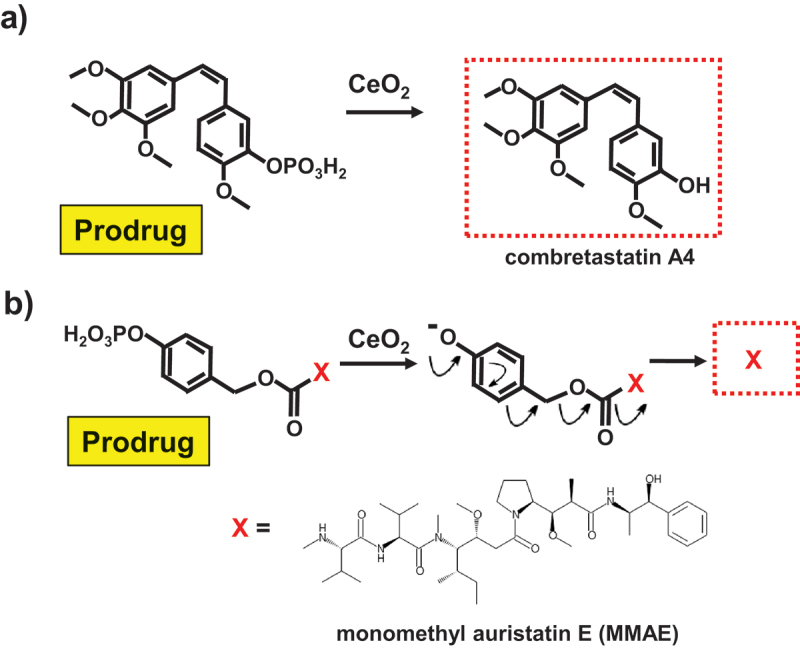
Dephosphorylation of prodrugs by CeO_2_ to release medicinally active drugs (combretastatin A4 in (a) and monomethyl auristatin E in (b)).

In pharmaceutics, prodrug strategy is widely used to improve the pharmacokinetic profiles of drugs that are unsuitable for direct administration. For example, water-insoluble drugs are often phosphorylated to increase the solubility and facilitate the administration into the body [171]. In the body, this phosphate is removed by alkaline phosphatase (and other phosphatases) to recover the original pharmaceutical activity. In Figure 9, CeO_2_ nanoparticles were used as artificial substitute of the phosphatases, and converted the phosphorylated prodrugs to the therapeutically active molecules [172]. In Figure 9(a), a phosphomonoester prodrug of water-insoluble anticancer agent (combretastatin A4) was converted into the parent drug, through the cleavage of the phosphomonoester by CeO_2_ particles as an enzyme mimic. When this prodrug was applied with CeO_2_ particles (1 g/L) to triple-negative human breast cancer cells (MDA-MB-231), the toxicity was notable.

In Figure 9(b), an amine-containing drug (monomethyl auristatin E for cancer therapy; X in red) is connected to 4-hydroxybenzyl alcohol via a carbamate linkage. In the prodrug used for the therapy, the phenolic OH in the resultant conjugate is further phosphorylated. In human cells, this phosphomonoester is hydrolyzed by CeO_2_ nanoparticles to induce 1,6-elimination reaction in the whole molecule. As a result, the drug (monomethyl auristatin E) is released with the liberation of CO_2_. Molecular design of these phosphorylation-based prodrug systems is flexible to allow versatile applications.

### 6.3. Decomposition of biofilms to facilitate antibacterial treatments

**Figure 10.**
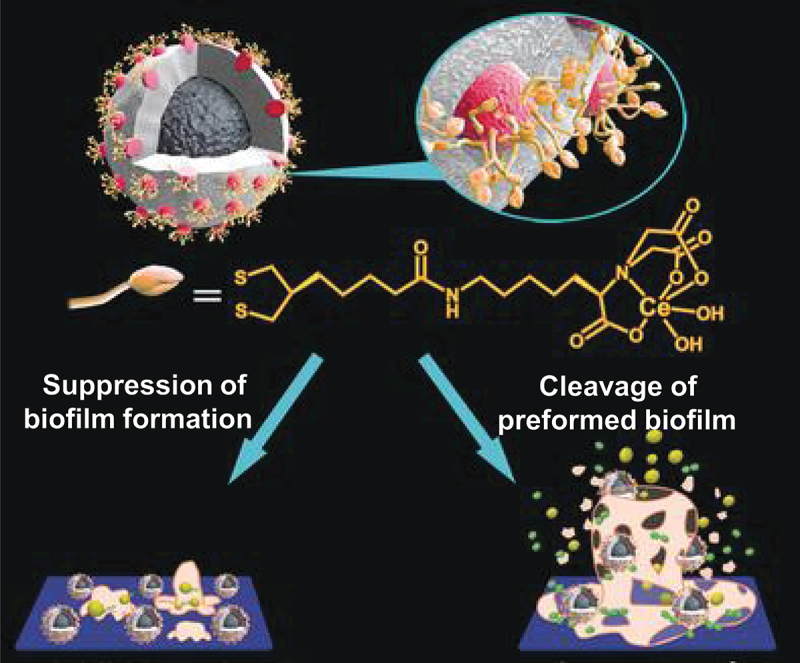
Ce^4+^-based composite to suppress the formation of bacterial biofilms. In the composite, Ce^4+^ complexes are bound onto the SiO_2_ layer of Fe_3_O_4_/SiO_2_ core/shell colloidal particles. By attaching the composite onto the surface of substrate, the formation of biofilms is suppressed through the cleavage of extracellular DNA by the Ce^4+^ complex (left). Furthermore, preformed biofilms can be dispersed by subsequent addition of the Ce^4+^-based composite (right). Reproduced from Ref. [177]. With permission from John Wiley and Sons.

Ce-based catalysts can suppress the formation of biofilms, which are produced by bacteria from extracellular DNA, polysaccharides, and proteins to protect themselves as shields [173–175]. These biofilms are a nuisance to medicinal treatments since they diminish the therapeutic effects of antibiotics [176] and also cover the surface of various medical devices. As a solution to these problems, Ce^4+^ complexes were bound onto the SiO_2_ layer of Fe_3_O_4_/SiO_2_ core/shell colloidal particles (light gray/dark gray particles in Figure 10) [177]. The target substrate of this composite is extracellular DNA which is one of the most important constituents of biofilms. The SiO_2_ shell was first modified with amino groups to bind citrate-capped gold nanoparticles (small red particles; 16 nm size). Then, an iminodiacetic acid derivative was covalently conjugated to the gold nanoparticles by chemical means. Finally, Ce(NH_4_)_2_(NO_3_)_6_ was added, and Ce^4+^ complexes were formed on the surface of core/shell colloidal particles. When the surface of the glass coverslip was coated with this Ce^4+^-based composite, biofilm formation by bacteria was effectively inhibited for a long period of time (bottom left). Furthermore, preformed biofilms of various ages can be dispersed by subsequently adding this composite to them (bottom right).

Another important application of catalytic cleavages of biofilms by Ce^4+^-based catalysts is to promote antibacterial effects of various therapeutic means through the removal of the shields of bacteria. For example, Ce^4+^ complex (to decompose biofilms) was bound to a MOF (to kill bacteria through peroxidase-like activity) [178]. First, Au-doped MOF was prepared from HAuCl_4_, FeCl_3_, and 2-aminoterephthalic acid. The Ce^4+^ complex of iminodiacetic acid was then bound to this MOF by a chemical procedure. The antibacterial effect of the composite is significant, since the Ce^4+^ complex first decomposed the biofilms to expose the bacteria for the attack by the peroxidase-mimic. Alternatively, photodynamic therapy is enhanced by coexisting Ce^4+^ catalysts, since reactive oxygen species (e.g. ^1^O_2_) can directly attack the bacteria without the protection of biofilms [179]. For example, nanoparticles containing meso-tetra(4-carboxyphenyl)porphine were first synthesized from this dye and ZrOCl_2_ and then mixed with Ce(NO_3_)_3_. Upon the incubation at 80°C, CeO_2_ nanoparticles were grown on the surface of a porphyrinic metal-organic framework (MOF). When bacteria-based diseases were treated with this composite, the biofilms covering the bacteria were first decomposed by the CeO_2_ nanoparticles through the cooperation of Ce^4+^/Ce^3+^ pair. Accordingly, reactive oxygen species, generated from the dye upon light irradiation, effectively killed the bacteria. In both cases, the therapeutic effects were remarkably increased through the predecomposition of biofilms. Chemotherapy was also promoted by CeO_2_-mediated biofilm destruction (6.4).

It was recently reported that the extracellular DNA as the main component of bacterial biofilms is left-handed Z-type DNA, rather than conventional right-handed B-type DNA [180]. With subtle perturbation by DNABII proteins of bacteria, B-DNA is converted to Z-DNA. This transformation is advantageous for bacteria to obtain nuclease-resistant biofilms since naturally occurring nucleases strictly distinguish between these two types of DNA and hydrolyze only B-DNA [181]. In contrast to strict preference of B-DNA by the enzymes, Ce-based enzyme mimics efficiently cleave both Z-DNA and B-DNA. Thus, versatile biofilms of bacteria can be easily decomposed by these mimics to promote many kinds of therapies.

In addition to the cleavage of extracellular DNA, CeO_2_ nanoparticles can also suppress the formation of bacterial biofilms through another mechanism in which these nanoparticles act as a mimic of another naturally occurring enzyme (haloperoxidase) [182,183]. There, hypohalous acid (HOCl and HOBr) is formed by the enzyme mimic from hydrogen peroxide and halide ions, and halogenates signal substances that are responsible for bacterial cell–cell communication.

### 6.4. Antibacterial and antiviral effects of Ce-based catalysts

Poly(acrylic acid)-coated CeO_2_ nanoparticles kill both Gram-negative and -positive bacteria very effectively because the phosphoesters in the cell membranes are destroyed by the CeO_2_ [184]. In a typical preparation of nanoparticles (10 nm size), Ce(NO_3_)_3_ and sodium polyacrylate were mixed in deionized water, and an ammonium hydroxide solution was added to the mixture. In the resultant nanocomposite, the CeO_2_ nanoparticles are coated with poly(acrylic acid) to show sufficient dispersibility. At pH 7.8°C and 37°C, this composite notably hydrolyzes the phosphodiester linkages in long-chain phospholipids (e.g. 1-palmitoyl-2-oleoyl-*sn*-glycero-3-phosphoglycerol) to induce eminent antibacterial activity. Importantly, this antibacterial activity of this composite is further promoted through the CeO_2_-mediated destruction of biofilms which cover the surface of bacteria (see 6.3). Thus, the CeO_2_-based composite successfully targets the cell membranes of bacteria, even when the bacteria are encapsulated inside the biofilms. However, it should be noted that antibacterial effects of CeO_2_ are never restricted to its phosphatase-like activity, and other mechanisms could be also responsible for these effects [185]. For example, CeO_2_ particles, adsorbed on the cell membrane, directly perturb the functions of bacterial proteins. Alternatively, Ce ions are released to alter electron flow, respiration, and other vital processes. Furthermore, reactive oxygen species are generated through Ce^4+^/Ce^3+^ redox reactions. Apparently, sufficient care must be paid when the antibacterial effects of CeO_2_ are analyzed.

Antiviral applications of Ce-based catalysts are also important. One of the currently important target viruses is SARS-CoV-2 (the virus of COVID-19), which caused a terrible pandemic from 2019 to 2023 [186–188]. Nanoparticles of CeO_2_ [189,190] and cerium molybdate (γ-Ce_2_Mo_3_O_13_) [191] showed high therapeutic activity. It was proposed that the Ce^3+^ ions in these drugs scavenge reactive oxygen species, which is produced in human cells in response to the viral infection and ultimately causes tissue damage [192]. However, another possible mechanism is that these Ce-based drugs directly destroy the genetic material of the virus (single-stranded RNA of 30,000 nucleotide-size). RNA is intrinsically far more reactive than DNA, and more easily hydrolyzed. Accordingly, this possibility would deserve detailed investigations.

### 6.5. Suppression of platelet aggregation

**Figure 11.**
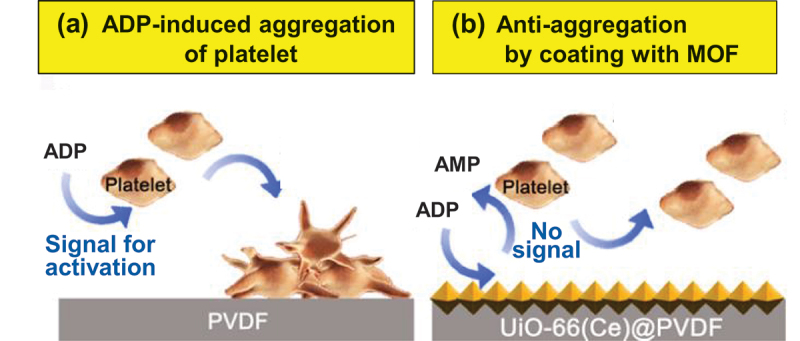
Suppression of platelet aggregation through the cleavage of ADP (activator of the aggregation) using Ce^4+^-based MOF. (a) Polyvinylidene difluoride (PVDF) surface without any modification and (b) the surface which is bound by the MOF. Reproduced from Ref. [153]. With permission from John Wiley and Sons.

In order to stop bleeding at injury sites, platelets aggregate each other through platelet-to-platelet adhesion and cover the sites. This essential bioprocess is initiated by the release of ADP from platelet-dense granules, which acts as a paracrine activator of platelets. Accordingly, the cleavage of ADP by Ce^4+^-based MOF in Figure 7 is employable to suppress this platelet activation and avoid unnecessary platelet aggregation for antithrombotic materials [153]. In Figure 11(a), platelet-rich plasmas of human blood are placed on the surface of a polyvinylidene difluoride (PVDF) film which is not pretreated by any means. The platelets are soon activated by extracellular ADP, and rapidly aggregate to adhere onto the film surface. On the other hand, platelet adhesion hardly occurs, when the Ce^4+^-based MOF catalyst is embedded onto the surface of the polymer film (Figure 11(b)). Apparently, the MOF cleaves extracellular ADP and blocks its interactions with platelets, as was observed with the use of the naturally occurring enzyme apyrase [193]. This anti-aggregation effect is applicable to antithrombotic coating on blood-contacting medical devices.

### 6.6. Clipping of desired fragments from huge genome by using Ce^4+^-based site-selective DNA cutter

Ce^4+^-based artificial site-selective DNA cutter, developed by the group of authors, is composed of both the Ce^4+^ complex of ethylenediaminetetraacetic acid (specific to the hydrolysis of single-stranded DNA) and a pair of peptide nucleic acids (PNAs) [84]. With the use of two PNAs, single-stranded parts are formed at the target site in a double-stranded DNA substrate, and selectively hydrolyzed by the Ce^4+^ complex. This artificial DNA cutter is applicable to homologous recombination in human cells [194–196]. Ce^4+^ complex can be replaced by nuclease S1. By extending this strategy, a useful tool to clip out a desired double-stranded fragment from huge DNA was developed [197,198]. The key is to attach a biotin to one of the PNA pair which is used to recognize the target scission site. In the clipping experiments, human genome is first digested to smaller fragments of appropriate sizes, which are then subjected to site-selective scission by an artificial DNA cutter (a combination of biotin-bearing PNA, another unmodified PNA, and the Ce^4+^ complex). After the site-selective scission, the target double-stranded DNA fragment is bound to the biotin-bearing PNA through Watson–Crick pairings. Accordingly, when the reaction solution is treated with streptavidin-coated magnetic beads, the target double-stranded DNA fragment is obtained in high purity. By developing a Ce-based solid catalyst showing single-stranded DNA specificity, the separation procedures can be further simplified for still wider applications.

## 7. Conclusion

Remarkable catalysis of Ce^4+^ ion for the hydrolysis of phosphoesters and pyrophosphates has been well evidenced. Its catalytic activity far exceeds those of other metal ions and non-metallic catalysts. Furthermore, this activity is greatly promoted by cooperation with other metal ions (e.g. Pr^3+^ and Nd^3+^). In these non-enzymatic systems, Ce^4+^ functions as a strong acid catalyst, whereas the second metal ion provides metal-bound hydroxide as an eminent nucleophile. This cooperation is reminiscent of the mechanisms of many enzymes for relevant reactions (alkaline phosphatase, purple acid phosphatase, and others). These fundamental results on liquid-phase catalysis have led to recent developments of Ce-based solid catalysts, which are significant from the viewpoints of both practical applications and mechanistic arguments. Typical examples are CeO_2_ and Ce-based MOF (metal-organic framework), which are satisfactorily active for the hydrolysis of various types of phosphate compounds. In these solid catalysts, the pairs of Ce^4+^ and Ce^3+^ ions are formed in the preparation processes, and cooperate for the catalysis.

As described in Section 2, phosphate compounds take inevitable roles in biology [199,200]. Thus, the catalysts presented here are important keys to regulate various bioreactions and their networks. Very straightforward idea should be to place these catalysts at predetermined sites in appropriately designed nanoarchitectures and construct novel biology/nanoarchitecture hybrids. The hydrolysis of phosphate can be an energy source to drive the hybrids, regulate molecular recognition in the hybrids, and provide the final output signal. When necessary, the activities of these catalysts can be increased by appropriate engineering, and their specificities are freely modulated. On-off regulation of catalytic activity by outer stimuli (e.g. photo-irradiation) is also possible. The applications of these interdisciplinary researches to medicine, molecular biology, environmental science, and many other fields are highly promising. In terms of still more sophisticated molecular design, the scope of their functions should be further greatly widened, providing unprecedentedly valuable tools for our future life.
